# The CIRCuiTS study (Implementation of cognitive remediation in early intervention services): protocol for a randomised controlled trial

**DOI:** 10.1186/s13063-018-2553-3

**Published:** 2018-03-15

**Authors:** Til Wykes, Eileen Joyce, Tjasa Velikonja, Andrew Watson, Gregory Aarons, Max Birchwood, Matteo Cella, Sue Dopson, David Fowler, Kathy Greenwood, Sonia Johnson, Paul McCrone, Jesus Perez, Andrew Pickles, Clare Reeder, Diana Rose, Swaran Singh, Dominic Stringer, Matthew Taylor, Rumina Taylor, Rachel Upthegrove

**Affiliations:** 1 Department of Psychology, Institute of Psychiatry, Psychology and Neuroscience, King’s College London, De Crespigny Park, London, SE5 8AF UK; 2 UCL Institute of Neurology, Queen Square, London, WC1N 3BG UK; 3 University of California, San Diego, 9500 Gilman Dr. (0812), La Jolla, CA 92093-0812 USA; 4 Mental Health and Wellbeing, Warwick Medical School, University of Warwick, Coventry, CV4 7AL UK; 5 Saïd Business School, University of Oxford, Park End Street, Oxford, OX1 1HP UK; 6 Psychology Department, University of Sussex, Brighton, Sussex BN1 9RH UK; 7 Sussex Partnership NHS Foundation Trust and University of Sussex, Sussex House, Falmer, Brighton, BN1 9RH UK; 8 Division of Psychiatry, UCL, Mental Health Sciences Unit, 2nd Floor Charles Bell House, 67–73 Riding House Street, London, W1W 7EJ UK; 9 King’s Health Economics, Institute of Psychiatry, Psychology & Neuroscience, King’s College London, De Crespigny Park, London, SE5 8AF UK; 10 Cambridge & Peterborough NHS Foundation Trust, CAMEO, Block 7 Ida Darwin, Fulbourn Hospital, Cambridge, CB2 5EE UK; 11 Department of Biostatistics and Health Informatics, Institute of Psychiatry, Psychology and Neuroscience, King’s College London, De Crespigny Park, London, SE5 8AF UK; 12 Health Services and Population Research Department, Institute of Psychology, Psychiatry and Neuroscience, King’s College London, De Crespigny Park, London, SE5 8AF UK; 13 Warwick Medical School, University of Warwick, Coventry, CV4 7AL UK; 14 South London & Maudsley NHS Foundation Trust, Maudsley Hospital, Denmark Hill, London, SE5 8AZ UK; 15 College of Medical and Dental Sciences, University of Birmingham, 25 Vincent Drive, Birmingham, B15 2F UK

**Keywords:** Cognition, Cognitive enhancement, Cognitive remediation, Cognitive training, Early psychosis, Implementation, Functioning, Psychological therapy, Recovery, Schizophrenia

## Abstract

**Background:**

Cognitive problems in people with schizophrenia predict poor functional recovery even with the best possible rehabilitation opportunities and optimal medication. A psychological treatment known as cognitive remediation therapy (CRT) aims to improve cognition in neuropsychiatric disorders, with the ultimate goal of improving functional recovery. Studies suggest that intervening early in the course of the disorder will have the most benefit, so this study will be based in early intervention services, which treat individuals in the first few years following the onset of the disorder. The overall aim is to investigate different methods of CRT.

**Methods:**

This is a multicentre, randomised, single-blinded, controlled trial based in early intervention services in National Health Service Mental Health Trusts in six English research sites. Three different methods of providing CRT (intensive, group, and independent) will be compared with treatment as usual. We will recruit 720 service users aged between 16 and 45 over 3 years who have a research diagnosis of non-affective psychosis and will be at least 3 months from the onset of the first episode of psychosis. The primary outcome measure will be the degree to which participants have achieved their stated goals using the Goal Attainment Scale. Secondary outcome measures will include improvements in cognitive function, social function, self-esteem, and clinical symptoms.

**Discussion:**

It has already been established that cognitive remediation improves cognitive function in people with schizophrenia. Successful implementation in mental health services has the potential to change the recovery trajectory of individuals with schizophrenia-spectrum disorders. However, the best mode of implementation, in terms of efficacy, service user and team preference, and cost-effectiveness is still unclear. The CIRCuiTS trial will provide guidance for a large-scale roll-out of CRT to mental health services where cognitive difficulties impact recovery and resilience.

**Trial registration:**

ISRCTN, ISRCTN14678860, Registered on 6 June 2016.

**Electronic supplementary material:**

The online version of this article (10.1186/s13063-018-2553-3) contains supplementary material, which is available to authorized users.

## Background

Schizophrenia is a relatively common disorder with a lifetime risk of around 1% [1]. It typically has an onset in late adolescence or early adulthood, so can derail the academic, interpersonal and employment achievements that prepare a person for adult roles and responsibilities [1]. It is also associated with an average loss of life-span of up to 20 years [2], poor employment prospects and difficulty in achieving satisfying social relationships. Poor prognosis is established soon after illness onset, with estimates of sustained social and occupational recovery being only 17–25% in the first 5 years [3]. Although positive symptoms (delusions and hallucinations) are a hallmark of a diagnosis of schizophrenia, cognitive dysfunction is apparent prior to the onset of psychosis and remains unchanged despite symptom remission [4, 5]. Poor cognition in people with a diagnosis of schizophrenia is a key predictor of poor functional outcome [6, 7] and impairments are noticeable in about 96% of all outpatients [8]. It is cognitive function at psychosis onset, and not symptom profile or response to treatment, that most strongly predicts social and occupational functioning 4 years later [9]. Cognitive difficulties also limit the rate of improvement using evidence-based rehabilitation, so that those who have the most difficulty will gain least [6]. Interventions that can boost cognition or maintain cognitive reserve would be beneficial, as these improvements are likely to have wide-ranging effects on service outcomes.

Owing to the potential for chronicity and morbidity, the economic burden of schizophrenia is immense. In the UK, it was estimated as £19b in 2012, and for each patient each year as £60k in societal costs and £36k in public sector costs (Schizophrenia Commission 2012 [10]) with similar figures found in the USA [11]. Much of the social burden is due to lost employment, housing and benefits [12]. New UK mental health policies, such as ‘No health without mental health’ [13], stress the need for early intervention to make long-lasting differences in people’s lives. With such a poor prognosis and high costs as well as personal burden, it is vital to explore whether new therapies can improve the recovery trajectory and thus decrease costs. Embedding cognitive treatments early, as in early intervention services (designed for clients who undergo intensive case management over the first 3 years of illness), may confer potentially long-lasting benefits.

Cognitive remediation therapy (CRT) was developed to address cognitive problems in people with schizophrenia. The Cognitive Remediation Experts Workshop in 2012 [14] defined it as ‘an intervention targeting cognitive deficit using scientific principles of learning with the ultimate goal of improving functional outcomes’ (p. 1). The largest meta-analysis (> 2000 participants in 40 studies) demonstrated that CRTs provide durable benefits in global cognition (effect size, 0.45) and functioning (Cohen’s *d* effect size, 0.42) [15] against any control group. New evidence and systematic reviews were taken into consideration by the Scottish Guideline Network for Healthcare Improvement Scotland [16] (extending the National Institute for Health and Care Excellence (NICE) guidelines of 2014 [17]) and CRT is now recommended in Scotland. Because of this wealth of evidence, other countries, such as Australia, Italy and Japan, and the New York State mental health services now include it in their guidance.

Cognitive remediation experts [14] recommend that ‘the effect on functioning is enhanced when provided in a context (formal or informal) that provides support and opportunity for extending everyday functioning’ p. 1. This is based on evidence that CRT boosted outcomes in other evidence-based therapies [18, 19]. One study, based in an early invention for psychosis services, also demonstrated that CRT can halve the number of cognitive behaviour therapy sessions needed for the same symptom reduction, reducing costs [19]. Early intervention services provide multimodal therapies, as well as contact with social and employment services. They therefore offer formal and informal opportunities for the translation of gains, as well as the potential to boost CRT outcome and improve the potential for changing recovery trajectories and sustaining benefits.

Cognitive remediation studies in younger people demonstrate acceptability and benefit in the short and longer term for cognitive and functional domains [20–22]; secondary analyses show greater gains for younger participants [23, 24]. There is ample evidence of biological and cognitive effects in schizophrenia, in which loss of brain grey matter [25, 26] and network disconnectivity occurs early in the disorder, but also evidence that CRT offers neuroprotective effects against grey matter loss [27] and improves brain activation [28]. As CRT has been shown to be effective for younger people and has the potential to improve functioning, it may be most beneficial if interventions are delivered at the earliest opportunity. There was optimism that early intervention services would have longer-term benefits but, despite quick access to multimodal treatments, it has been difficult to demonstrate that short-term improved outcomes were durable [29–31], although individual studies show better results [32]. On the whole, the results are like those of Bertelsen and colleagues [33] that, irrespective of receiving early intervention services, 60% of service users were neither working nor studying 5 years after psychosis onset. Clearly the current ingredients of recovery-focused treatments are not achieving their full potential for later function. This trial has also taken a recovery-focused approach and highlights those outcomes of relevance to an individual. Our primary outcome measure is, therefore, the important functional goal as chosen by the participant.

Cognitive remediation therapy is an evidence-based intervention but what is not obvious is the mode of implementation necessary and who would benefit most from different therapeutic modalities. Cognitive remediation therapies have been provided with high therapist involvement or very little, and at different intensity levels. We therefore developed the arms of the trial to represent the most frequently used implementation methods. The first is an intensive therapy that has previously been used by our team [34], which depends on continuous therapist support. The second arm is one adopted in many studies, where treatment is provided in a group with therapeutic support [35]. We have shown that our current therapy is suitable for such a group-intervention [36] approach. The final arm is one that depends on more independent access to therapy and has been used in several trials with differing effects [37, 38]. The main differences between these implementation methods is the level of therapist support and therefore the costs of implementation, with the most independent being the cheapest. Although the presence or absence of therapist support did not affect cognitive outcomes [14], therapist support has been shown to have tangible effects [39] and service users have positive views about therapists being present during therapy [40, 41]. Balancing the cost of the service, service user preferences and outcomes has not been tested as there have been no direct comparisons using the same cognitive remediation programme with differing levels of therapist support. The question of what is the best implementation method therefore has clinical equipoise. Cognitive remediation therapies have also generally been tested in single-centre studies so the effect of differing background services on outcomes has also not been tested. Therefore, this large, multicentre trial was developed, with the main aim of determining the best way of introducing CRT for psychosis into UK National Health Service (NHS) early intervention services in order to optimise individual functional outcomes and costs.

We consulted people with experience of using mental health services at every stage of trial development as well as clinicians and carers, mindful of the finding of Ennis and Wykes [42] that patient involvement is associated with study success. For example, clinicians do not routinely introduce the idea of cognitive difficulties at psychosis onset to service users. To address this sensitive issue, we consulted service users, carers and mental health clinicians through focus groups and developed three study leaflets, which were approved by an ethics committee, to accompany the participant information sheet. We also consulted the National Institute for Health Research Biomedical Research Centre Young Person’s Mental Health Research Advisory group about the design, wording of the protocol, participant information sheet, consent form and other promotional material for the trial.

### Trial aims and objectives

The aim is to determine the best method of introducing CRT for psychosis in UK NHS early intervention services to optimise individual functional outcomes and costs. The objectives are to compare three methods of CRT delivery on: (a) the degree to which participants have achieved their stated goals using the Goal Attainment Scale as the primary outcome measure; (b) improvement in cognition, social function, self-esteem and symptoms (the secondary outcome measures); (c) cost-effectiveness; )d) satisfaction of the service users and staff involved in the implementation.

## Methods

### Trial design

This is a multicentre, blinded, randomised, controlled trial conducted in the early intervention services of UK NHS Mental Health Trusts. Three different methods of providing CRT (intensive, group or independent) and treatment as usual within each of six research sites will be compared on their ability to improve real-world outcomes. In addition, implementation methods will be compared on their acceptability to service users, and the cognitive, clinical and real-world outcomes and cost-effectiveness, using a net-benefit approach. The site is designed to compare different services; catchment areas of the participating trusts range from high-density inner city to suburban and so cover diverse populations and different service backgrounds. Outcomes are measured at 0 (baseline), 15 (post-treatment) and 39 (follow-up) weeks after randomisation (see Additional file 1).

### Trial governance

The oversight of the trial is undertaken by a steering committee which, in addition to statistical and trial advisors, includes a service user and carer. In addition, an ECLIPSE service user advisory group meets three times a year to advise on any problems and to provide feedback on trial progress. The trial was registered at ISRCTN (ref.: 14678860), a primary clinical trial registry recognised by the World Health Organization and the International Committee of Medical Journal Editors. The trial was reviewed and given a favourable opinion by the National Research Ethics Service NHS Committee (Camden and King’s Cross Research Ethical Committee, ref. 15/LO/1960).

### Participants

Participants will be recruited from NHS early intervention services across six research sites in England (Birmingham, Coventry and Warwickshire, East Anglia, North London, South London, and Sussex).

#### Inclusion criteria

- Attending an early intervention service and at least 3 months from the onset of the first episode of psychosis; clinical stability, as judged by the clinical team
- Aged between 16 and 45
- Research diagnosis of non-affective psychosis, i.e. schizophrenia, schizo-affective or schizophreniform disorder following assessment
- Ability to give informed consent

These entry criteria were developed following discussion with staff, service users and carers to ensure a pragmatic approach. For example, the requirement for clinical stability will exclude a proportion of service users but this will be the approach used in reality to ensure that individuals can cope with the demands of the therapy, including regular attendance. It is also an entry criterion that both staff and service users recommended for the trial. The age criterion is the one adopted in early intervention services. The decision to consider individuals as potential participants after 3 months is based on the establishment of individuals into treatment in early intervention services following initial stabilisation after the acute episode.

#### Exclusion criteria

- Not able to communicate in English sufficiently to participate in cognitive testing
- Suffering from an underlying organic or neurological condition affecting cognition, e.g. traumatic brain injury or seizure disorder
- Have a comorbid diagnosis of intellectual disability

**Fig. 1:**
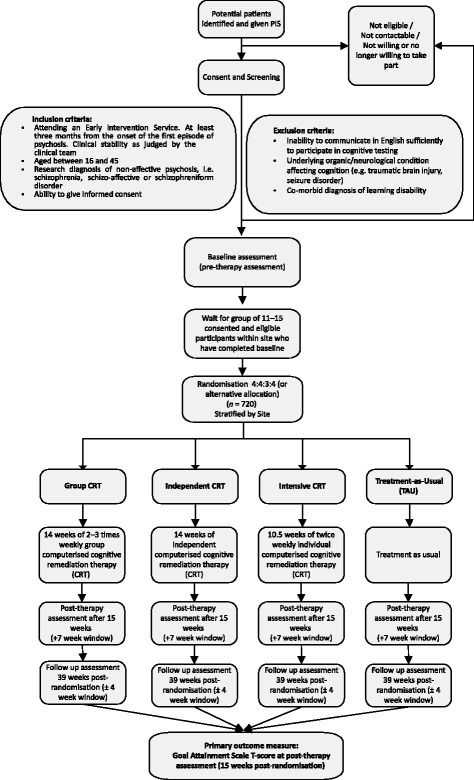
Participant flow chart. CRT, cognitive remediation therapy; PIS, patient information sheet; TAU, treatment as usual

**Fig. 2:**
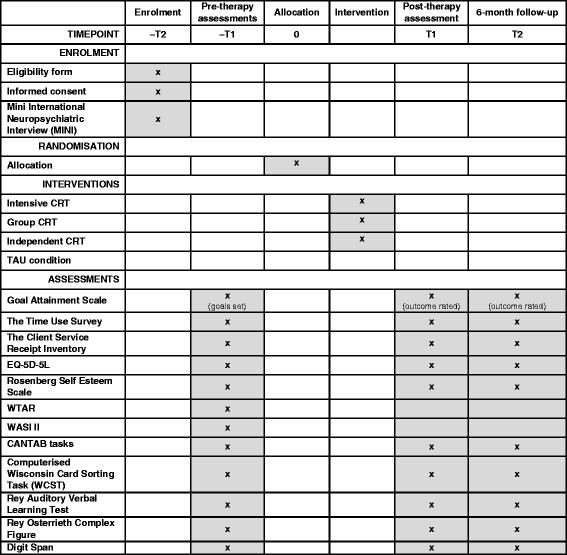
Schedule of enrolment, interventions and assessments. CANTAB, Cambridge Neuropsychological Test Automated Battery; CRT, cognitive remediation therapy; EQ-5D-5L, EuroQOL five dimensions questionnaire; MINI, Mini-International Neuropsychiatric Interview; TAU, treatment as usual; WASI II, Wechsler Abbreviated Scale of Intelligence – Second Edition; WCST, Wisconsin Card Sorting Task; WTAR, Wechsler Test of Adult Reading

At each site, the early intervention services clinicians will be asked to identify service users with a non-affective psychosis. Early intervention services clinicians will approach patients individually to ascertain permission for the research team to approach. Written consent will be obtained by trained research workers. After a consent form is signed, the researchers confirm the diagnosis by completing the relevant sections of the Mini-International Neuropsychiatric Interview (sections A ‘Major depressive episode’, D ‘Manic or hypomanic episode’ and L ‘Psychotic disorders’) [43]. Trial withdrawal will be recommended by the early intervention services clinician who provides treatment as usual. Figure 1 provides the participant flow chart; the enrolment schedule is provided in Fig. 2.

### Allocation and blinding

Therapists use secure email to send a list of (11–15) consenting and assessed participants to the King’s Clinical Trials Unit, which allocates each participant using pre-generated randomisation lists. The randomised allocations are sent back to the therapist by secure email. The pre-generated randomisation lists are stored by the King’s Clinical Trials Unit in an access-restricted electronic folder that is not accessible to any members of the study team. The therapist informs each of the participants of their allocated trial arm.

Randomisation is stratified by research site in the proportion 4:4:3:4 (Group CRT, independent CRT, intensive CRT, treatment as usual) to make efficient use of therapist resources. Alternative randomisation allocations will be used if 15 participants cannot be recruited within the stipulated 12-week recruitment period, with a minimum block of 11 participants (reducing the proportions in the independent CRT and treatment-as-usual arms only so as not to break the group design constraint or reduce the number of participants in the intensive CRT arm). This flexibility allows more efficient use of therapist time and resources as the therapist time is limited and the intensive therapy absorbs a lot of this resource.

The whole team, apart from the therapists and the randomisation statistician, are blind to participant group allocation. Breaches will be recorded; if a breach is to a research assessor, another research worker will complete the assessment. An audit of the quality of the blinding will be conducted at the end of the study.

Participant data are entered online into a secure electronic data capture system hosted by the King’s Clinical Trials Unit separately from treatment allocation data. Data quality and audit are regularly tested prior to and after data entry. Analysis scripts are agreed and finalised by the senior statistician prior to unblinding.

### Interventions

The CRT interventions (intensive, group, independent) will be carried-out using the CIRCuiTS computerised cognitive remediation programme; CIRCuiTS is based on a successful paper-and-pencil therapy and was developed with service users and therapists to increase engagement with younger clients who value computerised therapy [44, 45]. The three CRT delivery modes will differ in the associated hours of therapist contact but all participants will be offered 42 treatment hours. A treatment-as-usual arm will be evaluated, as CRT is not yet recommended in England by NICE; this will allow us to assess cost-effectiveness. All trial participants will continue to receive standard care throughout the trial.

Therapy will be delivered at each site by an experienced assistant psychologist trained in CRT at the trial centre and supervised centrally on a weekly basis. Each therapist will provide all three types of CRT over the therapy period.

- ***Intensive CRT***. Participants will receive 10.5 weeks of twice weekly therapy, up to 42 h in total, with sessions lasting between 60 and 180 min, split into three parts: (1) 20–60 min of CRT with a therapist; (2) 20–60 min of *in-vivo* transfer work (i.e. putting CRT strategies into real life) with a therapist; (3) 20–60 min of independent CRT, set up by the therapist on-site, or off-site in the service user’s own time.
- ***Group CRT***. Participants randomised in this arm will be offered 14 weeks of thrice weekly group therapy (up to 42 h of CRT). Group sessions will last up to 90 min, with attendance for at least 20 min considered as a completed session. Groups have closed membership, with four participants per group and one therapist. The sessions will begin and end with group activities, relating to goal setting and metacognition. During the rest of the session, service users will work independently on CIRCuiTS tasks, with the therapist offering help and support on an as-needed basis.
- ***Independent CRT.*** Participants will receive one individual session with the therapist for orientation followed by up to 41 sessions when they will work independently (up to 42 h of CRT in total). To support the independent sessions, the therapist will offer telephone contact or attendance at drop-in sessions on an as-needed basis to address any questions or problems (but not exceeding 1 h contact time per fortnight). A session will be considered ‘valid’ if it lasts a minimum of 20 min.
- ***Treatment as usual.*** This will be the standard input offered by the treating team without restrictions. Standard care involves clinical contact with the team on a daily, weekly or monthly basis depending on recovery. It also involves opportunities to be involved in educational or employment programmes, other psychological therapies, e.g. cognitive behaviour therapy for psychosis and medical treatments, including drug therapies. Participants randomised to the treatment-as-usual group will not receive CRT therapy.

### Outcomes

The ***primary outcome measure*** of the trial is the degree to which participants achieve their personal goals, as measured by the Goal Attainment Scale [46, 47] 15 and 39 weeks after randomisation. The Goal Attainment Scale is a method of scoring the extent to which participant’s individual goals (set at baseline) are achieved during the intervention. In effect, participants each have their own outcome measure but this is scored in a standardised way to allow statistical analysis. The goals are individually identified to suit the participant, and the levels are individually set around their current and expected levels of performance. The Goal Attainment Scale has been adopted in several studies of psychosocial interventions in mental health [48, 49]. It has been shown to be a reliable method of rating behaviours by self-report, which is comparable, but not identical, to informant and researcher reports, and has wide use in studies of cognitive rehabilitation and in clinical practice [50–52].

The ***secondary outcome measures***, some of which are used in the cost-effectiveness analysis, are: (a) social and occupational functioning, as measured by the Time Use Survey [53] and the EuroQOL five dimensions questionnaire [54]; (b) use of services, as measured by the Client Service Receipt Inventory [55]; (c) self-esteem, as measured by the Rosenberg Self-Esteem Scale [56]; and (d) cognition, as measured by the Cambridge Neuropsychological Test Automated Battery (which includes the following tests: Reaction Time, One-Touch Stockings of Cambridge, Paired-Associates Learning, Attention Switching Task, Rapid Visual Information Processing, Spatial Working Memory and Emotion Recognition Task) and supplemented by the Computerized Wisconsin Card Sorting Task [57], Rey Auditory Verbal Learning Test [58], Rey Osterrieth Complex Figure [59] and Digit Span forwards and back test [60]. We also collect some background data to investigate treatment mechanisms, including the Wechsler Abbreviated Scale of Intelligence [61] and metacognition measures. A description of these outcomes is presented in Additional file 2: Table S1.

Data are collected by trained research assistants, whose reliability is assessed regularly. Consistency between sites is achieved by regular review by members of the management and research teams as well as data quality checks at the site and by audit through the trial statistician.

### Measurement

#### Power

We have the capacity to recruit 900 patients (from 1500 patients attending 10 services for 3 years) and have allowed for a 20% drop-out pre-randomisation. Using a design with parallel arms of equal size, with 180 patients per arm, provides approximately 80% power for a simple group effect size difference of 0.3. This increases to 91% for outcomes that correlate 0.5 with baseline (both calculated using sampsi in Stata).

Freidlin *et al.* [62] suggest no great advantage in accounting for multiple testing in a multi-arm trial, and also that the advantages of a larger treatment-as-usual arm are more slight than commonly assumed. Interaction among patients in group delivery is very slight so no allowance for clustering was thought necessary.

The power calculation is based on arms of equal size; the difference in power as a result of the unequal allocation is likely to be small as the use of modestly unequal randomisation ratios only very slightly reduces the power of a study [63].

#### Analysis

***The primary outcome measure*** will be group differences in Goal Attainment Scale T-score [47] at 15 weeks post-randomisation, tested using an analysis of covariance (ANCOVA) model co-varying for Goal Attainment Scale T-score at baseline and adjusting for site as a fixed effect. Pairwise comparisons will be conducted between each of the CRT arms and treatment as usual, with significance and confidence intervals calculated using nominal values of *p*. Data will be analysed under intention-to-treat assumptions. Treatment effects for secondary outcomes will be analysed in a similar way. For the primary outcome, given the probable differences in treatment uptake (adherence), local average or complier average treatment effects compared with treatment as usual will also be estimated. Using assigned arm as an instrumental variable, all arms will be examined together to estimate the effect of hours of active CRT. On an assumption of a common per-hour effect across arms, some residual information will be available to estimate residual direct effects of treatment mode.

The ***cost-effectiveness*** analysis will be conducted from the perspectives of health and social care and society (including informal care, lost employment). Service use, collected using the Client Service Receipt Inventory, will be combined with appropriate unit cost information [64] and added to the intervention costs. Costs will be compared between groups using bootstrapped regression models to address the probable skewed distribution. Cost-effectiveness will be assessed by combining costs and outcome measures (primary outcome measure and quality-adjusted life years) in the form of incremental cost-effectiveness ratios.

If one arm has lower costs and better outcomes than another, it will be ‘dominant’. However, there will be uncertainty around the estimates of incremental costs and outcomes; this will be explored using cost-effectiveness planes and cost-effectiveness acceptability curves. The cost-effectiveness planes will be produced by generating and plotting (via bootstrapped regression models) 1000 incremental cost–outcome pairs. This will allow us to determine the probability that each arm has better outcomes and higher costs, better outcomes and lower costs, worse outcomes and lower costs, or worse outcomes and higher costs than the comparator. Cost-effectiveness acceptability curves will be generated using the net-benefit approach, whereby the incremental gain in quality-adjusted life years is multiplied by a range of threshold values for a quality-adjusted life year (including those used by NICE) and subtracting the incremental cost. This will be performed on 1000 bootstrapped incremental cost–outcome pairs and the proportion that are above zero will indicate that that one arm is more cost-effective than another. Sensitivity analyses will be conducted by varying key cost parameters. In particular, we will increase or decrease the intervention cost by 10%, 25% and 50% and use alternative methods for valuing informal care (e.g. *minimum* wage, unit cost of a homecare worker).

##### Interim analysis

Given that CRT is known to be effective, we want to ensure that we do not adopt all four trial arms if one treatment arm provides little benefit compared with the remainder, so we will carry out an interim intention-to-treat analysis. This will be undertaken by the health economist, using data from the first 195 patients (using the post-therapy data at 15 weeks post-randomisation). This analysis may result in one of the trial arms being closed, with an immediate impact on the randomisation of the next patients.

The decision to drop an arm will be taken by an independent data monitoring committee and will depend on the resultant cost of therapy and other services and goal attainment. The costs will include direct therapy inputs and other services derived from the Client Service Receipt Inventory.

The direct therapy costs will be calculated from data on the number and length of sessions, number of attendees (for group therapy) and unit costs, based on staff grade and overheads. Cost-effectiveness planes will be generated by plotting the 1000 incremental cost–outcome combinations for each pair of comparators. This will tell us the probability that one therapy has (i) lower costs and better outcomes, (ii) lower costs and worse outcomes, (iii) higher costs and better outcomes, or (iv) higher costs and better outcomes than a comparator.

### Governance and monitoring

The trial is sponsored by King’s College London, overseen by a National Institute for Health Research (NIHR) appointed ECLIPSE program steering committee, to which the trial’s independent data monitoring committee report. All members of the data monitoring committee are independent of the trial (are not involved with the trial in any other way and do not have competing interests that could impact the trial). The membership of all the committees can be found in Additional file 2: Table S2. The data monitoring committee is the only body involved in the trial that has access to the unblinded comparative data. It will receive and review the progress and accruing data of the trial and provide advice on the conduct of the trial to the trial steering committee. The role of its members is to monitor these data and make recommendations to the program steering committee on whether there are any ethical or safety reasons why the trial should not continue. Further details can be found in the data monitoring committee charter, which is based on DAMOCLES study group guidance [65]. Adverse events are reviewed by local principle investigators and stored locally. Serious adverse events are reported (emailed) within one working day to the local principle investigator and trial co-ordinator using password-protected forms. All serious adverse events are reviewed by a chief investigator to make a decision on whether or not they are definitely, possibly, or not related to the study intervention and whether they are expected or unexpected. All serious adverse events are anonymised and sent to a designated member of the data monitoring committee for their decision on whether they must be reported to the research ethical committee.

## Discussion

### Service user involvement

Service user involvement is an integral part of this study. Service users not only contributed to the study design but have also helped us develop the information sheets and consent forms and the publicity for the study, as well as advising us on how to approach potential participants. A service user and carer are also part of our steering committee. However, we have also chosen to meet our service user advisory group separately so that we can explain in more detail the issues we face and what our potential solutions might be. They can then provide advice that is not under time pressure or in the context of a large body of academics who may speak in jargon. This method of involvement has been suggested as important to ensure that service users feel they can provide worthwhile feedback [66]. Regular meetings are held to describe the recruitment, challenges and successes and the group is asked to advise on specific issues. The decisions of the user advisory group are then implemented and the minutes are available at meetings of the steering committee. Following advice on the effectiveness of user involvement [67, 68], we will ensure that our user advisory group provides value to our whole research programme by interviewing a sample of investigators each year to uncover and resolve any problems between the user group and the team. We will also ask the service user advisors to provide anonymous feedback on whether they think there are issues that have not been resolved satisfactorily or advice they feel that we ignored.

### Main challenges

The challenges fall into three areas: a changing context in the NHS; the availability of resources within teams; and block randomisation. Early intervention services have changed since the study was designed, as they now have specified waiting times and follow new NICE guidance on therapy packages. Both changes have affected how referrals are managed within teams. To overcome the new pressures, we will work closely with the clinical teams, team managers and care coordinators to ensure that the trial is not adding to these pressures. First, a full-time cognitive remediation therapist will be included in the early intervention team to provide early intervention staff an opportunity to refer their clients to CRT who might be on a waiting list for other therapy. Second, researchers will assist early intervention staff with risk-assessment reports (e.g. symptom assessments) and share a brief report on participant cognitive measures with the clinical teams, which will help them in their care programme approach for each individual, irrespective of whether they are involved in active therapy. Detailed cognitive assessments are usually not available in early intervention services, so this will be a benefit of choosing to take part in the trial.

Resources are always a difficulty, e.g. there may be a lack of suitable therapy rooms for CRT in some services, and we aim to help teams locate finance to re-use some unfurnished rooms. As CRT is not in NICE guidance, there is also a lack of experienced senior therapists who can provide specific supervision to more junior staff within a trust. We have responded by employing a senior clinical psychologist to offer this additional support across the sites and ensure continuity over the trial.

We developed our original block randomisation so that we could use the therapy resources as efficiently as possible and so blocks were defined as 15 participants. However, we potentially waste therapy resources with slow participant acquisition. Hence, we changed the blocks so that in some circumstances we can reduce the number of individuals who can be randomised. As with all trials there is a need to ensure blind assessment so we have also trained additional staff from each of the sites to provide extra support with some research procedures that might break the blind, e.g. inspecting clinical notes, and we will provide alternative raters in the case of any unblinding.

### Participant engagement and dissemination

Relevant research information will be available on the website, which is currently being developed (especially designed with participants in mind), as well as other social media (i.e. Twitter). This website will host presentations and peer-reviewed journal articles as they are produced to ensure accessibility. We will share our findings with our participants and participating teams through this method and also by sending newsletters at regular intervals to update them on the progress of the projects. Following advice from our user advisory group, participants will receive Christmas cards along with other promotional materials.

### Providing advice to the NHS

We are mindful that advice on implementation needs to come from different perspectives. Our three implementation models vary by therapist input and we will therefore have some detail on the effects on our key outcome variable, which is defined from the participants’ perspective – their goals as measured by the Goal Attainment Scale. But this is not all we will be measuring. We have the perspective of the service user participants on satisfaction with therapy and the method of provision, including therapy drop-out and the number of sessions received. We are collecting staff views so that we know what they consider appropriate levels of commitment and resource, as well as the organisational facilitators and barriers. We will include a provider perspective through the costs and cost-effectiveness of the different methods of providing therapy. All these perspectives will allow us to provide a balanced view of the different intervention methods so as to optimise their effects.

As well as a comprehensive overall plan for the best implementation method, our data will also allow us to discover whether therapy might need to be tailored to different individuals to provide the best effect. We will therefore investigate whether individual characteristics can predict larger or smaller benefits and, importantly, whether therapy might have a negative effect in some people. We will also investigate whether organisational factors, such as staff resources and background treatments, might affect successful CRT implementation.

All this information will allow: (i) policy makers to plan for this treatment; (ii) individual teams to understand what is required before and during implementation; and (iii) service users to receive the best individualised care to improve their recovery potential. Another of our work packages involves producing and evaluating an online training resource for this form of cognitive remediation. Together with the information on tailoring, this trial will allow smooth roll-out of the therapy into NHS services. Finally, the ECLIPSE programme will provide an implementation guide using the best available data.

## Trial status

Research protocol, version 1.3, 1 March 2017.

Recruitment start date, 1 June 2017; predicted recruitment end date, 1 January 2020.

## Additional files

Additional file 1: SPIRIT 2013 checklist. (DOCX 41 kb)

Additional file 2: Supplemental information. **Table S1:** Description of measures; **Table S2:** Membership of committees; Participant information sheet; Consent form. (DOCX 978 kb)

## Abbreviations

ANCOVA: Analysis of covariance
CIRCuiTS: Computerised Interactive Remediation of Cognition – Training for Schizophrenia
CRT: Cognitive remediation therapy
ECLIPSE: Building Resilience and Recovery through Enhancing Cognition and Quality of Life in the Early Psychoses
NHS: National Health Service
NICE: National Institute for Health and Care Excellence
NIHR: National Institute for Health Research
