# Supplementary material for: The CIRCuiTS study (Implementation of cognitive remediation in early intervention services): protocol for a randomised controlled trial
Source: Trials. 2018 Mar 15;19:183. doi: 10.1186/s13063-018-2553-3 (PMC5856221; doi:10.1186/s13063-018-2553-3)
Supplement: Supplementary file 2 — Supplemental information. Table S1: Description of measures; Table S2: Membership of committees; Participant information sheet; Consent form. (DOCX 978 kb) [file 13063_2018_2553_MOESM2_ESM.docx]

**Additional file 2**

**Table S1 Description of measures**

|  |  |
| --- | --- |
| **Measure** | **Description** |
| **Goal Attainment Scale** | This is a reliable method of rating behaviours by self-report which have been shown to be comparable but not identical to informant and researcher reports and has wide use in studies of cognitive rehabilitation and in clinical practice. GAS is calculated by first identifying goals through an interview with the service user to identify the main problem areas and establish an agreed set of priority goal areas. Then the goals are weighted on a four point scale. |
| **The Time Use Survey** | This is adapted for young people measures use of time compared to non-clinical age-matched samples from Oxford International Association for Time Use Research and the Office of National Statistics survey. Assessment captures a range of economic production and consumption activities that take place outside of the paid economy. It is sensitive to change and has good reliability |
| **The Client Service Receipt Inventory** | Versions of the CSRI have been used in around 400 studies in the UK and internationally. The aim is to record service use as comprehensively as possible (and feasible) over a specific retrospective period. In most mental health versions, services would include specific interventions received and inputs from primary care, secondary care and social care agencies. Data are mainly collected from those using services in the form of an interview but these can be supplemented from official records |
| **EQ-5D-5L** | The EQ-5D consists of five domains: mobility, self-care, usual activities, pain/discomfort, anxiety/depression. In the original version, each domain receives a rating from the patient of 1 (no problem), 2 (moderate problems), or 3 (major problems). This would usually take at most five minutes to complete. The subsequent five-figure score (i.e. between 11111 and 33333) is converted using an algorithm to a weight anchored by 1 (representing full-health) and 0 (representing death). This then allows the construction of quality-adjusted life years (QALYs) which are the favoured outcome measure of NICE. |
| **Rosenberg Self Esteem Scale** | This scale was originally devised as a measure for adolescents and young people. It consists of two scales, esteem and self-deprecation and has been used in several studies of CRT. It is feasible, shows good reliability |
| **CANTAB tasks** | |
| **Reaction Time** | **A 3 minute test measuring simple and 5-choice reaction time.** |
| **One-Touch Stockings of Cambridge** | **A** 10 minute problem solving test akin to the Tower of London in which participants are required to work out the number of moves required to make a display of coloured ball match a second display. There are a series of problems with different levels of difficulty. **Outcome measures** include the number of problems solved on first choice, mean choices to correct, mean latency to first choice and mean latency to correct. |
| **Paired Associates Learning** | **An** 8 minute test where participants learn and remember the location of up to 8 different patterns simultaneously. **Outcome measures** include the errors made by the participant, the number of trials required to locate the pattern(s) correctly, memory scores and stages completed. |
| **Attention Switching Task** | **An** 8 minute test of cognitive flexibility and rule learning with **outcome measuring** response latencies and error scores that reflect the participant’s ability to manage multitasking and the interference of incongruent task-irrelevant information on task performance. |
| **Rapid Visual Information Processing** | A 7 minute continuous performance test with several levels of difficulty measuring latency, probability of false alarms and sensitivity. |
| **Spatial Working Memory** | **A 4 m**inute **task measuring visuospatial working memory for the location of up to 8 identical targets and measuring** errors, strategy and latency. |
| **Emotion Recognition Task** | **A** 6 minute task where computer-morphed images derived from the facial features of real individuals, each showing a specific emotion, are displayed on the screen, one at a time. The participant must select which emotion the face displayed from 6 options (sadness, happiness, fear, anger, disgust or surprise). **Outcome measures** cover percentages and numbers correct or incorrect and overall response latencies, which can be looked at either across individual emotions or across all emotions at once. |
| **Supplementary Cognitive Tasks** | |
| **Computerised Wisconsin Card Sorting Task (WCST)** | A test of abstraction and cognitive flexibility measuring percentage errors |
| **Rey Auditory Verbal Learning Test** | A measure of verbal learning and memory |
| **Rey Osterrieth Complex Figure** | A test of visual memory for elements of a complex figure. |
| **Digit Span** | A verbal working memory task requiring brief retention and repetition of a series of digits either as presented or in reverse order. |
| **WTAR** | A brief reading test of words which cannot be pronounced phonetically and provides an estimate of pre-morbid IQ |
| **WASI II** | WASI-II provides a reliable, brief measure of IQ using 4 subtests of the WAIS IV. |

**Table S2 Membership of Committees**

| **Programme Steering Committee** | Professor David Kingdon (Chair)  Professor Graham Dunn  Professor Thomas Barnes  Marshall Whiting  Dr. Thomas Kabir | Prof Mental Health Care Delivery  Statistician  Psychiatrist  Carer  Public Involvement in Research Manager |
| --- | --- | --- |
| **Data Monitoring and Ethics Committee** | Professor Graham Dunn (Chair) Professor Anthony Morrison  Professor Richard Drake  Professor Rowena Jacobs | Independent Statistician  Clinical Psychologist  Psychiatrist  Health Economist |

**Participant Information Sheet**

**The CIRCUITS Study**

***This is a part of ECLIPSE Programme Grant (Building Resilience and Recovery through Enhancing Cognition and quality of LIfe in the early PSychosEs)***

We would like to invite you to take part in a research study. Before you decide you need to understand why the research is being done and what it would involve for you. Please take time to read the following information carefully and talk to others about the study if you wish.

Ask us if there is anything that is not clear or if you would like more information. Take time to decide whether or not you wish to take part.

**What is the research about?**

People who develop mental health problems may experience difficulties with cognition. Cognition refers to thinking skills. Thinking skills help people to remember information, plan, organise, problem solve and pay attention. For example, cognitive difficulties may mean you have to work harder to be with your friends or to keep your same standards in your daily life or at your job or school. But cognitive difficulties do not affect everyone to the same extent or in the same way.

Managing and adapting to these cognitive difficulties can lead to improved quality of life and better day to day functioning. Please see the attached leaflet which explains this in more detail.

We have developed a therapy known as Cognitive Remediation Therapy or CRT. This has been shown to help with thinking skills and everyday activities in our research studies. For example, some people regain much of their cognitive functioning through learning new strategies. Skills that you previously had may remain intact but need to be rediscovered. We now want to work out the best way to provide CRT in the NHS and specifically in early intervention services for psychosis.

The therapy we have developed works on a computer. The computer programme is called ‘CIRCuiTS’ and there are examples of how it works at the end of this document.

**Why are we asking for your help?**

You have been invited to take part in this study because you have had an episode of psychosis and are currently under the care of an early intervention service. During the course of the study approximately 700 people who have had psychosis will be asked to take part.

**Do I have to take part?**

It is completely up to you to decide whether you want to take part in the study. To help you decide, we will describe the study and go through this information sheet and the leaflet. We will also give you these to keep. If you decide to take part we will ask you to sign a consent form to show you have agreed. You are free to leave the study at any time, without giving a reason. This will not affect the standard of care you receive.

**Why are we doing the research?**

We already know that cognitive remediation therapy (CRT) is helpful but we want to know the best way to provide it.

In our trial, we will have four different conditions:

1. Cognitive-remediation therapy in a class with three other people;
2. Cognitive-remediation therapy as one-to-one with a therapist;
3. Cognitive-remediation therapy completed independently (on your own) with therapist support each week;
4. Treatment-as-usual, with no cognitive-remediation therapy.

If you agree to take part in the study you will be allocated to one of these four conditions described in detail below. The class therapy will be held on three days each week and will last an hour. One-to-one therapy is provided in two sessions per week and each session is longer – up to 3 hours. In the more independent therapy we expect participants to do three sessions of an hour each week on their own but with therapist support available weekly if needed. Usually the courses last for 14 weeks but the more intensive course lasts for 10 weeks.

**What will I have to do?**

If you agree to take part, there will be a number of stages to go through:

1. **Measuring cognition**. This will take place at the beginning of the study, at the end of the study and at follow-up (6 months later). You will be asked to complete tasks which measure your thinking skills, such as memory, problem solving, attention and organisation.

We also wish to assess ‘metacognition’. This is about how well you think you did on the tests. After two of the tests of cognition, we will ask you to answer some questions about how you think you did on the tests. We would like to record your responses using the iPad to make sure we collect all of this information.

In one assessment you will be asked to copy a complex figure. We would like to video your drawing using the iPad. You will not be identified in the video, as the camera will be focused on your hands/drawing only.

Finally, we will ask you to fill in two questionnaires about your thinking skills in general.

You will be guided in this process by a member of the research team who will be present to explain the procedures and answer any questions.

1. **Measuring your mental health and how you spend your time**. This will take place at the beginning, at the end of the study and at follow-up (6 months later). Using well-known questionnaires, we will ask you questions about your current symptoms (for example hallucinations or delusions), how they affect your every-day life and how much health and social care you receive.

At the beginning of the study we will also collect other relevant information on how you were doing before you experienced psychosis.

1. We will then ask you EITHER to **take part in a course of cognitive-remediation therapy** in addition to your normal treatment OR **continue with the treatment you normally receive** without having cognitive remediation therapy. This is so we can compare the different methods for cognitive remediation therapy with usual care.

Therefore, you will be allocated to one of the 4 groups (described above) at random, which will be done by the computer. If you are allocated to take part in cognitive remediation therapy, you will be assigned to one of the three types of therapy (class/one-to-one/independent). We are doing this to be able to understand which type of therapy suits early intervention services best.

As there are three possible types of therapy you will have a 75% chance of being allocated to cognitive remediation therapy that may help your thinking skills and everyday activities.

We are also going to ask you for a separate permission to look at your clinical notes to collect additional information such as: sociodemographics, clinical history, list of medications and any physical illnesses. If you consent to this part of the study, only researchers directly involved in the study will have access to your clinical notes.

**What are the possible disadvantages to taking part?**

There are no immediate disadvantages to taking part. You will receive the same regular care whether or not you agree to take part.

**What are the possible benefits?**

You will be randomly allocated to either one of the groups which will be receiving a therapy that may help your thinking skills and everyday activities, or you will continue with the treatment you normally receive. The cognitive-remediation programme will also follow your personal goals set up with the therapist at your first session.

If you decide to take part in the study you will help with the development of a new service for people with psychosis.

**What if I have other commitments and/or do not have time to take part in the trial?**

We realise that taking part in the trial might require a lot of time on top of your other commitments. Therefore, we will try to support you in any way possible to make the necessary arrangements.

**Will I be paid for taking part?**

You will not receive any payment for taking part in the therapy, but you will be reimbursed £7 an hour for your time doing the research interviews and cognitive tasks (taking place at the beginning, at the end of the trial and at follow-up 6 months later). For example, we have estimated that the first assessment (before the start of the trial) will take 3 hours 45 minutes, which means we would give you a total of £28. At the end of the therapy, the research interviews and cognitive tasks will last approximately 3 hours and at follow-up approximately 2 hours 30 minutes.

**What if there is a problem?**

The study is being led by Professor Til Wykes at the Institute of Psychiatry, Psychology and Neuroscience (Kings College London) and Professor Eileen Joyce at the Institute of Neurology (University College London). The study is being held at several different early intervention services across the country. There is a team at each of these sites with a therapist, a researcher and a consultant psychiatrist. Your researcher is (INSERT NAME for RA, email, number). Please contact him/her if you have any concerns about any aspect of the study.

If you remain unhappy, particularly about the way you have been treated during the study, then you can contact the study lead in XXX Trust (INSERT NAME for PI, email, number). You can also contact **Professor Til Wykes** (email: til.wykes@kcl.ac.uk) or **Professor Eileen Joyce** (email: [e.joyce@ucl.ac.uk](mailto:e.joyce@ucl.ac.uk)).

If you wish to complain formally, you can do this through the NHS Complaints Procedure. The telephone number and the address for you nearest patient Advice and Liaison Service (PALS) can be obtained from the hospital.

**Will my taking part in the study be kept confidential?**

Yes. All the information collected about you during the course of the research will be kept strictly confidential and any information that is stored will have your name and address removed so that you cannot be recognised. All identifiable data (such as consent forms, email addresses) will be kept in locked filing cabinets, separately from the other research data. Any direct quotation of the study participants will be anonymised. Only researchers directly involved in the study will have access to the passwords or keys.

We will also let your GP know that you have taken part in the study, but they will not have access to any of your data.

As a matter of good practice in clinical studies we would like to share a general report about some assessments to your clinical team. This is to help them with the planning of your care. You are also entitled to receive a copy of this report.

**What will happen if I don’t want to carry on with the study?**

If you decide to withdraw from the study at any time, you can do so, without expressing any reasons. It will not affect the care you receive. The data already collected however will be retained.

**What will happen to the results of the study?**

We intend to publish the results in journals where it will have the most influence. So we will publish in medical journals as well as making sure that service users will have access to the information whether or not they took part in the research. You will not be identified in any of these reports.

**Who is paying for this research?**

The research is paid for from the NHS as part of a grant from the National Institute for Health Research in England.

**Who has reviewed this study?**

All research in the NHS is looked at by an independent group of people called a Research Ethics Committee in order to protect your safety, rights, wellbeing and dignity. This study has been reviewed and given a favourable opinion by the NRES NHS Committee XXXX_XXXX.

This is a **homepage of CIRCuiTS** (computer cognitive-remediation programme), where you sign-in with the user log-in details provided to you. Only you and your therapist will be able to see the exercises you have been doing, follow the progress etc.


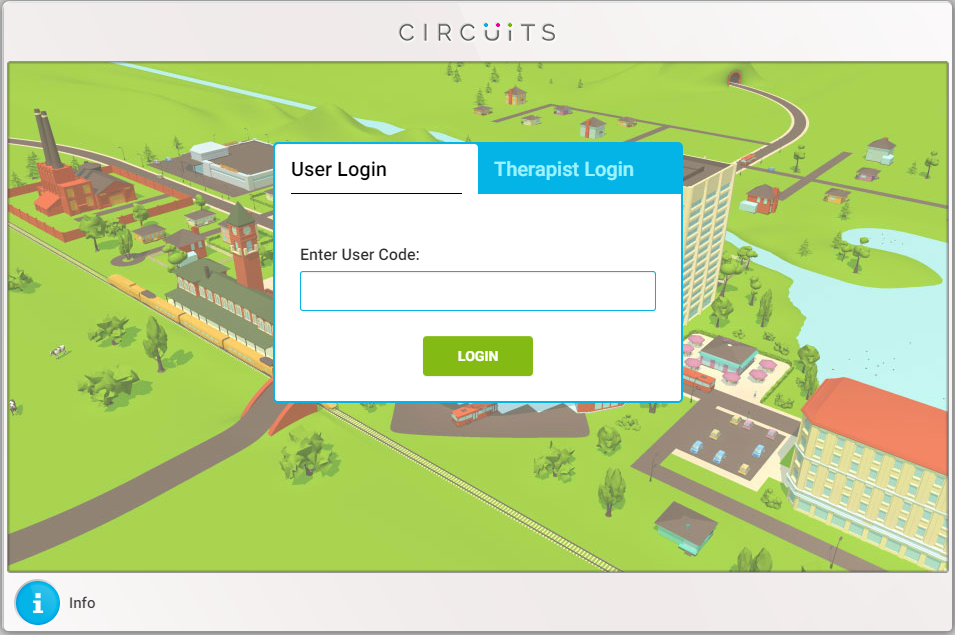


This is an example of CIRCuiTS (computer cognitive-remediation programme) **progress book**, where you can switch between different exercises, check the progress you have been making, review your goals etc.


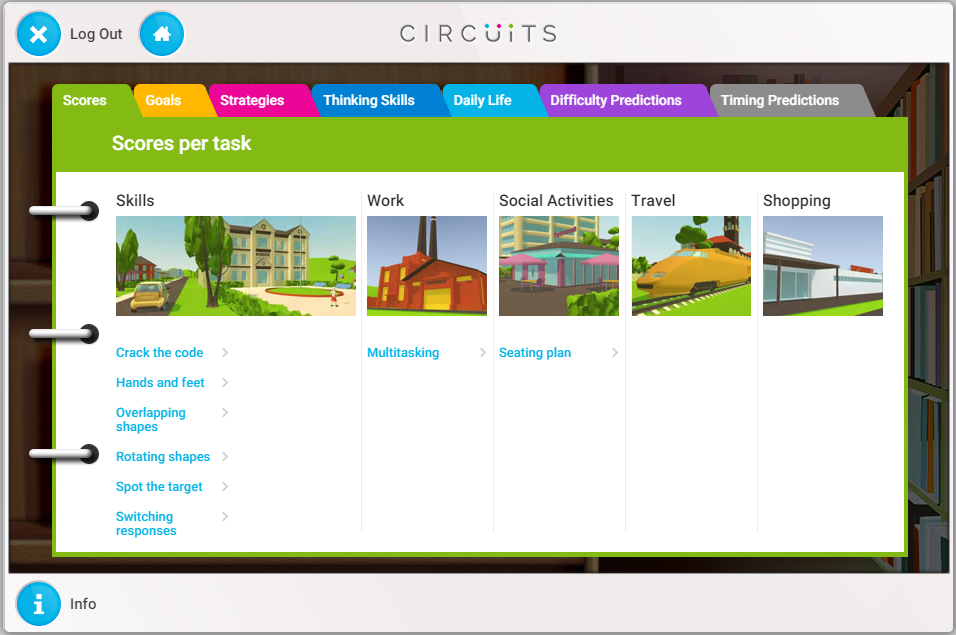


This is an **example of CIRCuiTS task**, intended to practice particular thinking skills. In this task you are asked to copy the image on the left hand site, using the ‘tools’ provided on the right hand site. This is more an abstract task, but some other tasks will also be based on real-life activities.


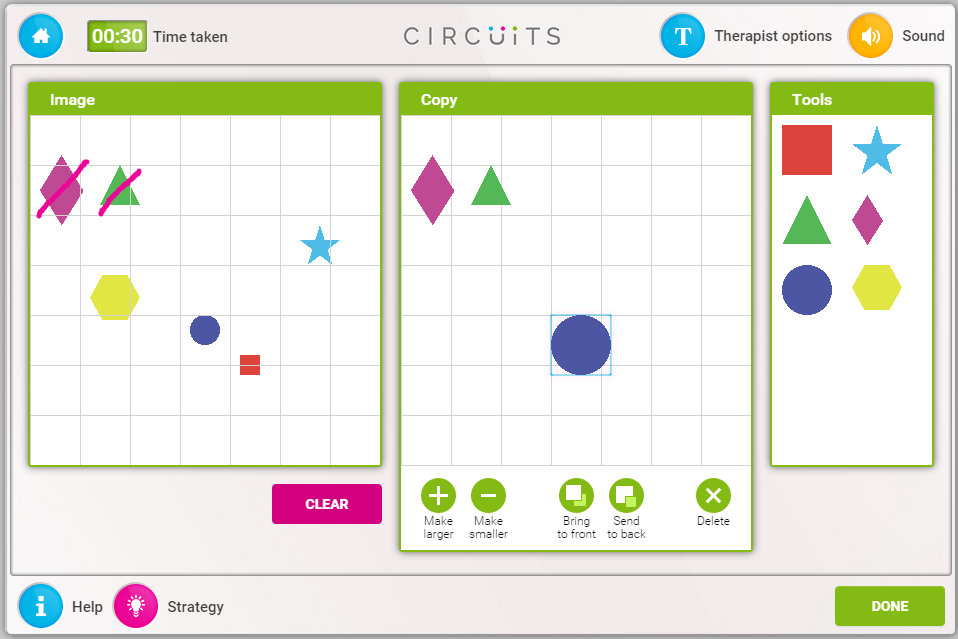


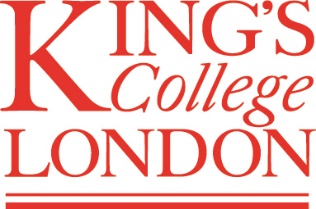

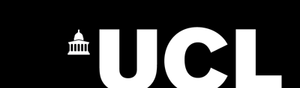


**[insert Trust Header]**

**Consent Form**

**Enhancing Cognition and quality of LIfe in the early PSychosEs (ECLIPSE)**

**Implementation of Remediation into Early Intervention Services**

The study lead for this study in XXX Trust is (NAME):email. xxx@xxx.

Please put your

initials into the box

1. I confirm that I have **read and understood** the information sheet dated 23^rd^ December 2015 (Version 1.1) for the above study. I have been able to think about the information. Any of my **questions have been answered** fully. I understand why the research is being done and the **risks involved**.

1. I understand that taking part **is voluntary**. I am **free to leave the study at any time** without giving any reason and without my medical care or legal rights being affected.
2. I understand that the data the researchers collect will be **stored anonymously** (during and after the study). This means that the data will not be able to be traced back to me.
3. I give permission for people in the research team to **look at my clinical notes** and analyse them in strict confidence. People that are outside of the research team will only have data that they cannot trace back to you. These procedures comply with the Data Protection Act.
4. I give permission for people in the research team **to contact my GP** or other medical services to collect data regarding my use of those services.
5. I agree that the information collected about me can be examined and **stored for up to 7 years** at the study sites. This is in accordance with Medical Research Council data handling guidelines.
6. I understand that I can contact the study co-ordinator to **look at the data collected** about me in this study. I am allowed to change or delete any of this data if I feel that it is incorrect.
7. I am happy for a **GP** and my **Care Co-ordinator** **to be informed** that I have taken part in this study.
8. I **agree** to take part in this study.

------------------------------------ ------------------------ -----------------

Name of participant Signature Date

------------------------------------ ------------------------ -----------------

Name of Person taking consent Signature Date

**Would you like to be sent any further information (including any research outcomes) about the study in a newsletter?** (Please circle) **YES** *Email:*  **NO**

When completed:

1 copy is for the participant.

1 copy is for the research file.

1 (original) is for the medical notes.
